# Congenital syphilis in Switzerland: a marker of inequality? A mini-review

**DOI:** 10.3389/fpubh.2023.1265725

**Published:** 2023-09-14

**Authors:** Antoine David, Klara M. Posfay-Barbe, Carina Aguiar Nogueira, Laurence Toutous Trellu

**Affiliations:** ^1^University Hospitals of Geneva, Geneva, Switzerland; ^2^Maternidade Escola Assis Chateaubriand, Fortaleza, Brazil

**Keywords:** congenital syphilis, maternal syphilis, congenital syphilis/prevention and control, congenital syphilis management, syphilis epidemiology, inequality, healthcare access, mother to child transmission

## Abstract

Syphilis remains a global public health problem, with growing incidence in most regions of the world, particularly among women of childbearing age. This alarming trend has led to an increase in cases of congenital syphilis, resulting in devastating consequences. While the implementation of measures by the World Health Organization (WHO) and various governments has contributed to a decline in the global incidence of congenital syphilis, many countries are facing an escalating crisis, as incidence continues to rise. This mini-review aims to provide an overview of the current state of this disease in different parts of the world, focusing on the most affected populations and highlighting congenital syphilis as a marker of vulnerability. It also focuses on Switzerland, a country with a robust economy, to identify shortcomings in the healthcare system that contribute to the persistence of congenital syphilis, even though the infection is easily detectable and treatable. In conclusion, this mini-review highlights the persistent risk of congenital syphilis worldwide, regardless of country prevalence or economic status, and underscores the need for sustained efforts to reach underserved women, emphasizing the vital role of comprehensive training for healthcare professionals.

## Introduction

The number of syphilis cases remains very high worldwide, including in Switzerland; this disease mostly affects women of childbearing age (1, 2). Due to the high risk of mother-to-child transmission, with an estimated risk of transplacental transmission of up to 80% (3), and because of the availability of effective and accessible treatment, it is universally recommended that all pregnant women be screened for syphilis, regardless of their risk-taking behaviours, or pregnancy history (4–8). Furthermore, prenatal screening and treatment are effective in preventing congenital syphilis, even in countries where the prevalence of syphilis is low (9, 10). Pregnant women should therefore be screened at least during the first and third trimesters, as well as at delivery if they exhibit at-risk behaviors. In several countries, these repeated routine screenings are unconditionally recommended (3, 11–23).

The purpose of this review is to examine the epidemiological situation across various regions of the world, and to explore some of the factors that contribute to the continuing public concern about congenital syphilis. We performed a literature research. We analyzed monocentric and multicentric, national and international studies published the last two decades in English language on Medline, as well as newsletters and websites of governmental and non-governmental organizations. Key words were congenital syphilis, maternal syphilis, vulnerability, mother-to-child transmission, and name of each continent.

## Management

Treatment of pregnant women for primary, secondary, or early latent syphilis consists of one intramuscular injection of 2.4 million units of penicillin G, and, for late latent or undetermined onset of infection, of three similar injections at one-week intervals (4). Alternative antibiotics must be avoided: macrolides do not cross well the fetoplacental barrier and tetracyclines are contraindicated during pregnancy. Resistance to these classes of antibiotics has also emerged in recent years. In cases of allergy to penicillin, desensitization is recommended (24–26). According to the World Health Organisation’s (WHO) estimations, correct treatment of a pregnant woman with syphilis can reduce fetal or neonatal mortality, as well as stillbirths by 80%, and the risk of prematurity or low birthweight by 65% (27). Treatment of sexual partners is also crucial, as there is a risk of reinfection of well-treated pregnant women (28).

Treatment of newborns is indicated if congenital syphilis is detected or strongly suspected, if the mother has not been treated, or if she was treated less than 4 weeks before delivery. Treatment consists of 100,000–150,000 units per kg of aqueous benzyl penicillin given intravenously daily for 10–15 days. Alternatively, intramuscular treatment of 50,000 units per kg per day of procaine penicillin G is recommended for 10–15 days (4, 29, 30). After treatment, follow-up begins at the first month of life, then at 2, 3, 4, 5, 6, 12 and 24 months of age. Usually, the non-treponemal test (RPR/VDRL) becomes negative around 6 months of age.

**Figure 1:**
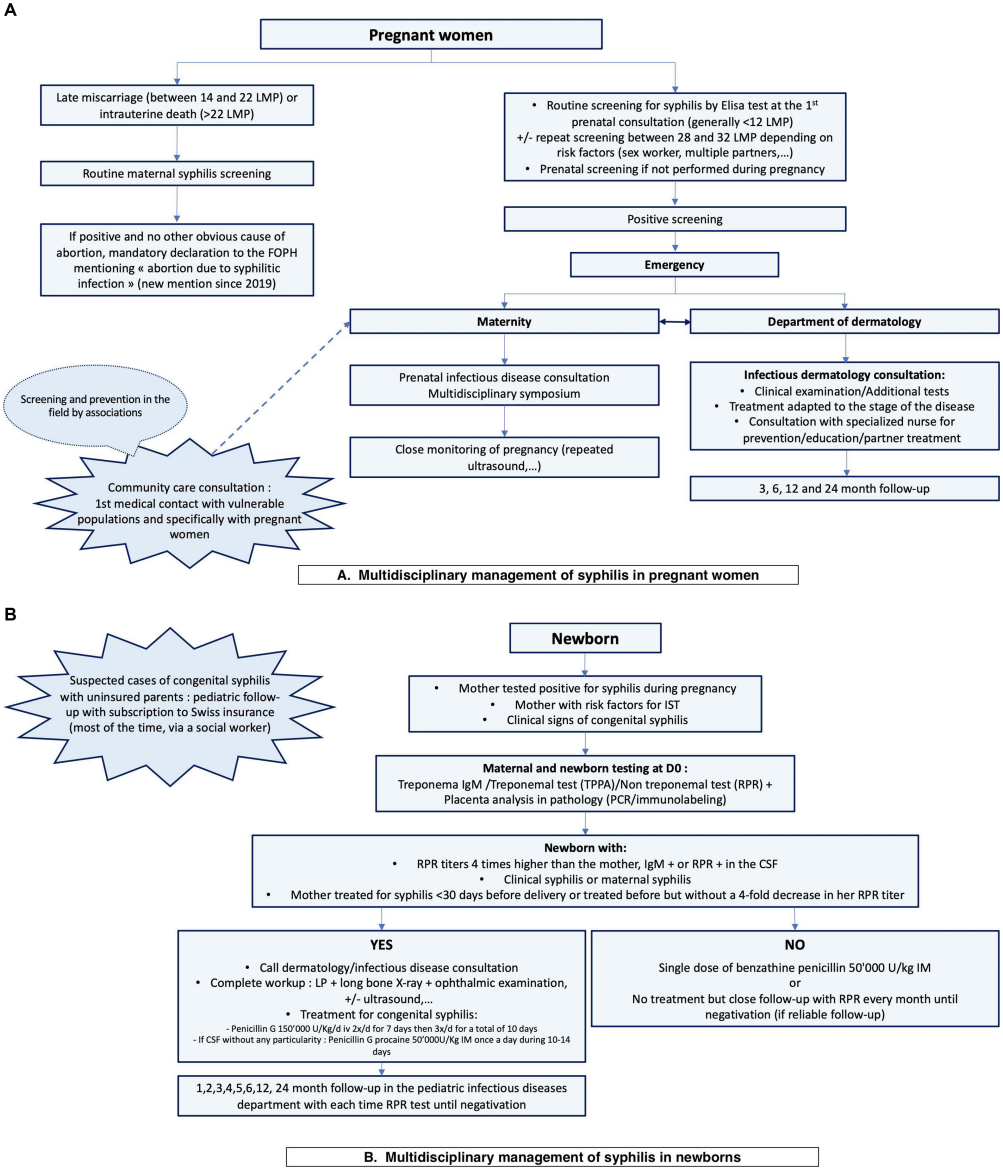
Institutional flowchart of the multidisciplinary management of syphilis in pregnant women **(A)** and their newborns **(B)**. LMP, weeks of last menstrual period; FOPH, Federal Office of Public Health; CSF, cerebrospinal fluid; LP, lumbar puncture.

To optimize the management of cases, a multidisciplinary approach is necessary. At Geneva University Hospitals, we use the same flowchart to harmonize practices and to propose appropriate treatment for mother and child in the post-delivery context (Figure 1).

## Congenital syphilis: situation over the past few years

### Alarming situations around the world

In 2007, the WHO set the goals of eradicating congenital syphilis with an incidence of less than 50 cases per 100,000 live births, as well as providing prenatal screening and adequate treatment, if necessary, to at least 95% of pregnant women (9, 27). The aim is to achieve this in 80% of all countries by 2030. Between 2012 and 2016, the incidence of syphilitic infections in pregnant women remained virtually unchanged, with a WHO estimate of 700 and 690 cases per 100,000 pregnant women, respectively. Between 2012 and 2016, the estimated global incidence of congenital syphilis also decreased from 539 to 473 cases per 100,000 live births. This 12% decrease may reflect a progress; however, the rates are still far from the target set by the WHO (27).

Although the incidence is mostly decreasing worldwide, it is unfortunately not the case everywhere. In the United States, the rate of reported congenital syphilis has increased sharply since 2012 (11, 12). In fact, there has been a 477% increase between 2012 and 2019, with the incidence of congenital syphilis rising from 8.4 to 48.5 cases per 100,000 live births (31). Four possible explanations are the parallel increase in primary and secondary syphilis cases in young women of childbearing age, which almost tripled between 2015 and 2019 (3, 12, 13), the lack of prenatal screening and adequate treatment, as well as syphilitic reinfections during pregnancy (13, 32, 33). In fact, in 2019, 60% of congenital syphilis cases in the United States resulted from a lack of prenatal screening, or from inadequate treatment of pregnant women with established diagnoses (12). In addition, several studies observed considerable racial and ethnic disparities, with higher rates found among minorities and vulnerable populations, such as Native populations, African Americans or Hispanic individuals (3, 12–14). Some studies also observed higher rates in economically disadvantaged populations, drug users, or individuals released from incarceration (13, 15).

Similar observations were reported in neighboring countries, including Canada, with a 10- to 15-fold increase of congenital syphilis between 2016 and 2020 (16). Once again, the populations identified as most at risk were Native American and economically challenged patients: they face a lack of access to care, longer delays between diagnosis and treatment with many missed opportunities (16, 34). Mexico was also affected by a 6-fold increase in the number of congenital syphilis cases between 2010 and 2019 (17).

In South America, each country uses its own definition of congenital syphilis and notification type may vary. Cases are probably underestimated (35). Brazil is particularly affected, with an alarming increase from 143 to 861 cases per 100,000 live births between 2002 and 2016 (19); a slight decrease to 770 cases per 100,000 live births was nevertheless reported in 2020 (36). This large number of cases can be explained by the lack of access to rapid tests or treatment in certain regions (36). Despite the extent of Brazilian health system and community participation in the health sector, there is still insufficient prenatal screening and many missed opportunities with an increase in untreated or inadequately treated cases (19, 37). A study conducted in a hospital in the North East (Conceiçao) between 2008 and 2018 showed that 61% of pregnant women diagnosed with syphilis received inadequate treatment or were not treated at all (20). Similarly to North America, indigenous populations remain the most affected (36).

Other South American countries, such as Paraguay, raise concerns, with an incidence of 197 cases per 100,000 live births in 2018 according to reports; however, the WHO estimates a 9-fold discrepancy between estimated and reported cases, with an estimate of 1792 cases per 100,000 live births. A 1.6-fold increase in cases between 2018 and 2020 has been noted (38).

Concerning developing and emerging countries, the African continent remains the most affected area, with over 60% of the worldwide congenital syphilis cases between 2012 and 2016. This can be explained by the high maternal prevalence and the lack of access to antenatal care (6, 27).

Elsewhere, developed countries such as Japan, Australia and New Zealand have also described outbreaks of congenital syphilis, with an incidence of 9.4 cases per 100,000 live births in the latter between 2018 and 2020 (39). In Japan, the incidence is also gradually increasing (from 0.4 to 1.4 cases per live birth between 2012 and 2016), raising a public health concern (18). Again, the most at-risk populations include the most vulnerable populations (drug users, etc.) (17, 39). In Australia, it has been described that the incidence of congenital syphilis is up to 20 times higher in the indigenous populations than in non-indigenous populations (40).

However, there are also countries with declining incidence rates. In China, the beginning of the millennium (2000–2011) was marked by an almost 25-fold increase in congenital syphilis cases, prompting the country to implement an action plan to improve screening and treatment of the disease, doubling the number of pregnant women screened for syphilis between 2011 and 2018. During the same period, the incidence of congenital syphilis decreased significantly from 91.6 to 18.4 cases per 100,000 live births; however, the number of syphilis cases diagnosed in pregnant women continues to increase each year (41, 42). This example shows that implementing a national action plan can be effective.

Finally, the only continent to have reached the goal set by the WHO for the elimination of the disease is Europe (27). The number of congenital syphilis cases reported in the European Union has decreased overall since 2005, despite an increasing curve in recent years, according to ECDC’s online interactive Surveillance Atlas of Infectious Diseases (21, 22). Indeed, in 2018, for the first time since 2013, the number of reported cases increased, with 60 confirmed cases of congenital syphilis in 23 European Union countries, representing an incidence of 1.6 per 100,000 live births (22, 43). The latest available data date from 2021, with an incidence of 1.8 cases per 100,000 live births (44). These data illustrate the impact of measures taken by governments in prenatal screening programs for the disease; covering 98% of pregnant women in 2016–2017, the European Union had the highest rate in the world (1). However, data are still underestimated, and underreporting is highly probable (21, 22). On a smaller scale, there is a wide variation among European countries, with incidence ranging from 0.1 to 39.8 cases per 100,000 live births in some years (22, 23). Eastern European countries seem to be the most affected, especially Bulgaria, with an incidence of 20 cases per 100,000 live births recorded in 2016. In the same year, the second and third place were held, respectively, by Poland and Romania (21). In Bulgaria, the number of cases continued to increase with a 250% rise between 2017 and 2019 (17). Once again, the most vulnerable populations, such as migrant women, sex workers and drug users, are mostly affected; social marginalization and exclusion delay access to care (8, 22, 45).

### Case of Switzerland

In Switzerland, the incidence varies greatly from year to year. Between 2013 and 2022, it varied from 0 to 8.14 cases of syphilis per 100,000 inhabitants under the age of 1, with an average incidence of 1.6 over the last 10 years (2). The notification system has been modified in recent years: notably, stillbirths and abortions associated with syphilis infection have been notified since 2019. Physicians notify stillbirths and abortions in a woman with positive syphilis test for which no other etiology was found (46). However, the epidemiological data are not very detailed and may be underestimated since congenital syphilis is reported in the same form as all syphilis. It has been shown that 20% of pregnant women in Switzerland have not been screened for syphilis (47). Compared to the general Swiss population, studies have reported that undocumented migrants present later to antenatal care and have a higher risk of sexually transmitted infections in the context of risky behavior (48). A 10-year study at the University Hospital of Zurich has shown that among nine pregnant patients with a positive treponemal test, eight were immigrant patients (49).

It is important to understand where the failures of a developed country’s healthcare system like Switzerland come from, why certain vulnerable populations are left out, despite theoretical measures to include them.

## Swiss healthcare system and its limitations

### Case of undocumented migrants and asylum seekers

Because of its strong socio-economic position, Switzerland is exposed to both legal and illegal migration. It is estimated that Switzerland has up to 100,000 undocumented migrants: many are middle-aged women from Latin America, Eastern Europe, Asia and North and West Africa (48).

There are three main groups of migrants in the country:

- “Legal” migrants, who received a residence permit, a health insurance, and have the same access to the Swiss healthcare system as the general Swiss population. They usually have no problem accessing healthcare.
- Asylum seekers who are registered and distributed in all the Swiss cantons (states). In the same way as the other citizens, asylum seekers have a health insurance. They generally have access to healthcare like all other residents. Nevertheless, language barriers exist.
- Undocumented migrants are defined as people living in Switzerland without a valid residence permit. Considered residents in Switzerland (50), they must apply for health insurance within 3 months after taking up residence in Switzerland; after giving birth, they must obtain insurance for their child within the same period. They can also benefit from reduced insurance costs, and insurers cannot refuse a registration request. However, many migrants do not apply for insurance coverage for economic reasons.

The response of the Swiss healthcare system varies greatly, with different policies for each canton.

Only 2 of the 26 Swiss cantons (Geneva and Vaud) have established primary care services for undocumented migrants, providing free or inexpensive access to health facilities. In the other cantons, these primary care services are provided by non-governmental organizations or, in some cases, exist only in the emergency departments of public hospitals (48, 51).

A study of the undocumented migrant population in the canton of Geneva shows that only 16% are covered by required health insurance (52).

In a situation of distress, the Swiss Constitution guarantees that patients receive essential care and are treated in a manner consistent with human dignity, regardless of their insurance cover (53).

Despite the theoretical measures, the Swiss healthcare system has many limitations for undocumented migrant patients.

### Limitations of the Swiss healthcare system for these minorities

First, we note a cost barrier. In Switzerland, health insurance fees and co-payments are very high, which sometimes leads to insurance coverage being waived, care being forgone, or treatment being delayed.

Additionally, undocumented individuals may also fear being reported and avoid contact with public institutions as much as possible. In case of non-payment, despite medical confidentiality, insurers try to recover the money, which increases the risk of being discovered.

Furthermore, migrants are likely to be isolated and face language barriers, resulting in a lack of information on access to healthcare in general, despite the work of some associations that carry out medical prevention at strategic points.

Another difficulty that can be encountered by migrants is filling out administrative documents. The administrative difficulties to obtain health insurance are considerable: insurance companies often make it difficult for them to take out a policy, with refusals (due to lack of knowledge of the law) or unfeasible requests (e.g., a postal address or a residence permit). In addition, the language barrier increases the difficulty of understanding administrative documentation and procedures.

Finally, although emergency care is provided, chronic health problems requiring continuity of care on an outpatient basis are not necessarily covered in all cantons (48, 51).

## Discussion

Congenital syphilis is a severe and fatal disease that can be easily prevented and cured. The alarming incidence rates reported in all parts of the world, both in resource-limited and developed countries, highlight the undeniable weaknesses of the healthcare systems in terms of accessibility and quality of care. Regardless of the economic situation of countries, the most affected populations remain the most vulnerable minorities, notably native populations, or undocumented migrants. In these communities, access to healthcare can be complex and people may avoid consultations and wish to keep discretion. As a result, the risk of transmission rises due to inadequate disease management.

Developing countries face a lack of resources that contributes to these shortcomings, sometimes with remote areas having no access to screening or adequate treatment. In developed countries, undocumented individuals have economic, social, and legal barriers that are particularly difficult to overcome, despite legislation offering access to healthcare for all. Regardless of the economic status of the country, mechanisms commonly found are the banalization of the disease by the general population, a lack of awareness among healthcare providers, which can lead to missed diagnoses or inadequate treatment, as well as a lack of care for partners, which often leads to reinfection during the pregnancy. In 2021, a review of the *Archives of Disease in Childhood* proposed replacing the acronym for congenital infections TORCH (toxoplasmosis, others, rubella, cytomegalovirus, herpes simplex) with SCORTCH (syphilis, CMV, others, rubella, toxoplasmosis, chickenpox, HSV and blood born virus) to include congenital syphilis (54).

More global obstacles to success can be identified, such as the regular shortage of benzathine penicillin, particularly in more than 40 countries between 2014 and 2016 (19, 20, 22, 55), which sometimes leads to the use of therapeutic alternatives that do not cross the fetoplacental barrier. It should be noted that the treatment is still not produced in Switzerland, despite several initiatives from organizations, such as the Federal Office of Public Health (FOPH) (5). Moreover, since regular errors in prescribing or administering medication have been observed in all countries, we would like to re-emphasize in this review that a good knowledge of treatment (penicillin formulations and dosages) is the foundation of infection control.

The development of multiple action plans at global level through the WHO, but also at a national level, has already shown its effectiveness, as seen in China. However, much remains to be done in most parts of the world, especially in the Americas and on the African continent. Although Africa is the continent most affected by congenital syphilis, it is the least represented region in the various studies in the international literature.

At the Swiss level, a retrospective and prospective study supported by the Swiss National Science Foundation is underway. It aims to quantify the number of cases of congenital syphilis and syphilis during pregnancy in Switzerland, and to describe their characteristics and risk factors. A national register should then be integrated to target women at risk during pregnancy and optimize the follow-up of babies who test positive.

## Conclusion

The epidemiological overview of congenital syphilis shows a persistent risk of babies being born with asymptomatic or severe syphilitic infection in many countries. Even in developed countries with efficient healthcare systems, numerous challenges remain in reaching certain underserved women. Ongoing training of healthcare professionals is crucial, not only in areas with a high prevalence of disease, but also in regions where cases are rare, and where neglecting the infection may also impact lives.

## Author contributions

AD: Conceptualization, Writing – original draft. KP-B: Supervision, Validation, Writing – review & editing. CA: Validation, Writing – review & editing. LT: Conceptualization, Supervision, Validation, Writing – review & editing.

## Funding

The authors declare financial support was received by the Swiss National Science Fundation (SNF), grant number CRSII5 186394, for publication of this article.

## Conflict of interest

The authors declare that the research was conducted in the absence of any commercial or financial relationships that could be construed as a potential conflict of interest.

## Publisher’s note

All claims expressed in this article are solely those of the authors and do not necessarily represent those of their affiliated organizations, or those of the publisher, the editors and the reviewers. Any product that may be evaluated in this article, or claim that may be made by its manufacturer, is not guaranteed or endorsed by the publisher.
